# Bioactive Potential of Arazá (*Eugenia stipitata*) Seeds: Hypoglycemic, Antiradical, and Nutritional Properties

**DOI:** 10.3390/plants14111662

**Published:** 2025-05-30

**Authors:** Claudia Cristina Pérez Jaramillo, Jonh Jairo Méndez Arteaga, Liceth N. Cuéllar Álvarez, Walter Murillo Arango

**Affiliations:** 1 Grupo de Investigación en Productos Naturales, GIPRONUT, Universidad del Tolima, Ibagué 730006, Colombia; ccperezj@ut.edu.co (C.C.P.J.); jmendez@ut.edu.co (J.J.M.A.); 2 Grupo de Investigación en Productos Naturales Amazónicos-GIPRONAZ, Facultad de Ciencias Básicas, Universidad de la Amazonia, Florencia 180001, Colombia

**Keywords:** *Eugenia stipitata*, byproduct utilization, antiradical activity, phenolic compounds, glycemic enzyme inhibition

## Abstract

Arazá (*Eugenia stipitata*) seeds, which are an abundant byproduct of pulp processing in the Amazon region, represent up to 84% of the fruit’s dry matter and remain underutilized. This study investigates, for the first time, the bioactive potential of hydroethanolic (70:30) extracts from Arazá seeds (ASs) to inhibit key enzymes related to glycemic and cholesterol regulation, specifically *α*-amylase, *α*-glucosidase, and HMG-CoA reductase. Additionally, the proximate characterization, antioxidant capacity assessment, and LC-MS analysis of phenolic compound composition were performed. The results demonstrated that the hydroethanolic extracts exhibited the significant inhibition of *α*-amylase and *α*-glucosidase, with IC_50_ values of 47.06 and 49.99 µg/mL, respectively. This inhibitory activity correlates with the total phenolic content (155.88 ± 6.12 mg GAE/g dry weight) and compounds such as epicatechin gallate and *p*-hydroxybenzoic acid. The extract also showed a high capacity to scavenge the DPPH radicals (IC_50_ = 46.63 µg/mL), although no inhibition of HMG-CoA reductase or cytotoxicity in blood cells was observed. Proximate analysis revealed that ASs are low in lipids (0.16%), proteins (4.96%), and ash (0.82%) but contain a considerable amount of fiber (27.7%). These findings suggest that ASs represent a valuable byproduct with potential for further research on its application in diabetes management.

## 1. Introduction

Arazá (*Eugenia stipitata*), a fruit native to the Peruvian Amazon rainforest, is cultivated in Brazil, Peru, Ecuador, Bolivia, and Colombia. This fruit is distinguished by its high levels of vitamin C, dietary fiber, and minerals, such as iron and calcium [1]. Arazá has emerged as a potential resource for improving socioeconomic conditions in the western Amazon region due to its attractive sensory characteristics and nutritional components, which may contribute to the well-being of those who incorporate it into their regular diet [2]. However, its consumption is limited to local markets, with low levels of exports. The fruit is not typically consumed fresh but is processed and exported as frozen pulp in various presentations, especially from Costa Rica and Colombia. The focus on processing the fruit into pulp results in the seed becoming the major byproduct, representing 30% of the fruit’s volume, 22% of its fresh weight, and 84% of its dry matter [3]. It is estimated that approximately 220 kg of seed is produced per ton of processed fruit. This makes the seed a valuable resource to consider in processes related to the integral utilization of the fruit’s biomass, potentially leading to biorefineries based on the use of the whole fruit. Despite this potential, studies on the nutritional and bioactive potential of ASs, mainly organic extracts focused on controlling diseases related to metabolic syndrome, such as hyperglycemia and hyperlipidemia, are scarce. This syndrome, which is a significant public health concern in the 21st century, is characterized by a cluster of metabolic abnormalities, including abdominal obesity, high triglycerides, low HDL cholesterol, high blood pressure, and high blood sugar. It significantly elevates the risk of type 2 diabetes, cardiovascular disease, and other comorbidities, such as nonalcoholic fatty liver disease, cholesterol gallstone disease, and reproductive disorders [4]. Numerous studies have investigated the underlying mechanisms of metabolic syndrome, its relationship with various comorbidities, and the potential of lifestyle interventions, including the incorporation of Amazonian fruits, to prevent or manage these conditions [5]. Recent studies have shown that ASs are a significant source of minerals, including calcium, magnesium, and potassium, as well as fiber and phenolic compounds, such as simple phenols and, predominantly, flavonoids, including apigenin, catechin, quercetin, and luteolin, which are found in higher proportions than in the fruit pulp.

This study aims to comprehensively analyze and compare the chemical and nutritional composition of ASs with that reported in the published literature. Furthermore, it is the first study to evaluate the antioxidant, hypoglycemic, and hypocholesterolemic activity of seed extracts. This, along with the other biological activities analyzed, can serve as a basis for understanding the potential of this byproduct and developing strategies to utilize it as a source of active compounds of interest in the control and prevention of chronic non-communicable diseases related to metabolic syndrome.

## 2. Results

### 2.1. Proximate, Mineral, and Chemical Composition of ASs

Arazá seeds (ASs) are a byproduct of fruit processing, constituting approximately 84% of the fruit’s dry matter [1]. This substantial portion of the fruit’s total biomass remains largely unutilized yet holds potential value for food applications or as a source of bioactive compounds. Table 1 presents the proximate and chemical composition of ASs. It reveals a significant fiber content (27.7%), while lipid (0.16%) and protein (4.96%) levels are low, suggesting that ASs are not a primary source of these nutrients. The ash content is minimal, although it offers a diverse range of minerals.

The total sugar content and the levels of total phenols and flavonoids in ASs are lower and higher compared to those of the fruit pulp, respectively, which is consistent with previous research [6,7].

### 2.2. Phenolic Composition and Antiradical Capacity of ASs

Table 2 shows the results for the determination of phenolic compound levels in ASs, where seven phenolic compounds were quantifiable. Epicatechin gallate (EGCG) was the most abundant (2.3 mg/kg), followed by p-hydroxybenzoic acid (1.1 mg/kg). During the commercialization of Arazá, the fruit pulp is primarily used; however, it cannot be ruled out that the seed (an agro-industrial byproduct) is a potential source of bioactive compounds, as evidenced by these results. The authors of [8] studied a mixture of these fractions, reporting phenolic compound contents of 42.81 ± 2.23 and 41.59 ± 6.26 mg/100 g and flavonoid contents of 2.52 ± 0.25 and 2.52 ± 0.30 mg/100 g for aqueous and methanolic extracts, respectively. They also quantified various phenolic compounds, including caffeic acid, cinnamic acid, p-coumaric acid, (−)-epicatechin, ethyl gallate, ferulic acid, gallic acid, quercetin-3-glucoside, and vanillin, in the aqueous extract. The authors of [6] reported the identification of phenolic compounds in Arazá pulp and seeds using ESI-LTQ-XL-MS/MS. Notably, the seed fraction (ASs) exhibited the highest number of identified compounds, particularly in positive ionization mode, including cinnamic acid, gallic acid, and, predominantly, flavan-3-ols, flavonones, and flavones. However, it is important to note that these identifications are based on relative intensities, which were observed to be low for the reported phenolic acids.

The antiradical capacity of ASs was assessed using DPPH assays and expressed as the inhibition percentage. The non-linear regression analysis models generated from the data are shown in Table 3. Based on these models, the IC50 was estimated to be 46.64 µg/mL, with a 95% confidence interval (CI) of 40.98–53.70 µg/mL.

### 2.3. Biological Activities and Chemical Composition of the AS Hydroethanolic Extract

**Figure 1:**
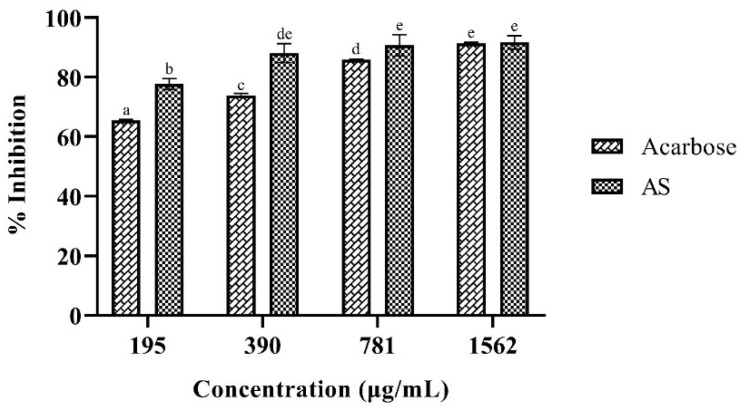
*α*-Amylase inhibition at different concentrations of AS hydroethanolic extract. Different letters indicate statistically significant differences (*p* < 0.05).

**Figure 2:**
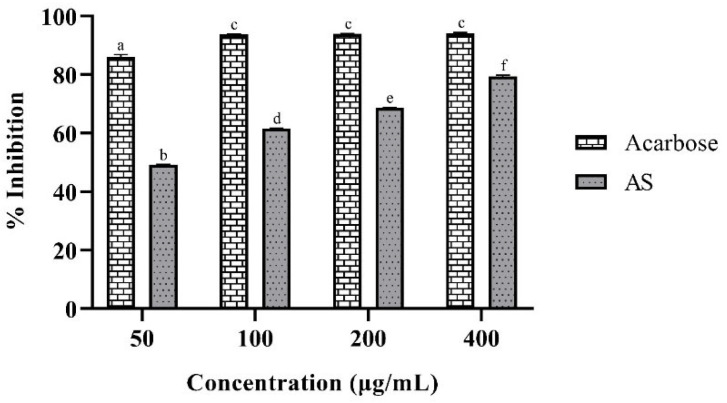
*α*-Glucosidase inhibition at different concentrations of AS hydroethanolic extract. Different letters indicate statistically significant differences (*p* < 0.05).

As shown in Table 2, seven phenolic compounds were identified in the AS hydroethanolic extract, with epigallocatechin gallate (EGCG) being the most abundant, followed by p-hydroxybenzoic acid. Figure 1 illustrates the relationship between the concentration of the AS hydroethanolic extract and the percentage of *α*-amylase inhibition, which is well-fitted to the non-linear model shown in Table 3. The IC50 calculated from these data was 47.06 µg/mL, with a 95% confidence interval (CI) of 33.42–60.55 µg/mL. The IC50 value of the AS hydroethanolic extract (Table 3) was lower than that of acarbose (82.88 µg/mL), demonstrating a nearly two-fold higher inhibitory potency. However, the inhibition of *α*-glucosidase was lower than that achieved with acarbose at the same concentrations. Figure 2 shows the inhibition behavior of this enzyme at different concentrations for both acarbose and the AS hydroethanolic extract. The respective calculated IC50 values (95% CI) were 49.99 µg/mL (39.98–61.81 µg/mL) and 12.46 µg/mL (9.17–15.73 µg/mL).

As previously mentioned, the identified phenolic compounds in the AS extracts exhibit radical scavenging, antiradical, and *α*-amylase and *α*-glucosidase inhibitory activities. However, the significant inhibition of HMG-CoA reductase was not observed, with only 17.29 ± 0.01% inhibition achieved.

## 3. Discussion

The results of the proximate analysis and other nutritional compounds were lower than those reported by [6], who showed fiber, lipid, and protein values of 33.74%, 1.03%, and 5.95%, respectively. While the ash content was comparable, the mineral composition differed. The reference study reported higher concentrations of minerals such as K (231.99 mg/100 g d.w), Ca (22.37 mg/100 g d.w), Mg (35.80 mg/100 g d.w), and Na (54.15 mg/100 g d.w). Based on these data, the Na/K ratio can be calculated. In our case, this ratio was significantly greater than 1, while in the study [9], it was less than 1. Maintaining a Na/K ratio below 1 is crucial for normal blood pressure regulation [9]. Furthermore, the authors of [10] reported 28.96% extractives, 91.10% total solids, and 3.99% volatile solids for ASs, indicating potential for extract production, the exploration of bioactive compounds, and the utilization of seeds for biogas generation. Likewise, the carotenoid content in ASs was substantial, measured at 43.66 ± 1.5 mg β-carotene/100 g dry weight. While no previous study has reported the carotenoid levels within ASs specifically, the pulp and peel have been documented to contain 62.85 ± 1.92 µg β-carotene/g and 24.84 µg/100 g dry weight, respectively [11]. However, the paucity of comprehensive research on ASs limits a thorough comparative analysis of their chemical and proximate composition. This is particularly relevant given the significant impact of agroclimatic factors in different growing regions, which influence overall fruit composition. Future research should prioritize addressing these factors to better understand ASs. Our results demonstrate a lower total phenol content than that reported in [1] for AS samples from the Colombian Andean region but a higher total phenol content than that reported in [6]. Furthermore, the results of the antiradical capacity are probably attributable to the high phenolic and flavonoid content previously reported for extracts obtained using organic solvents and water [6,10]. The antiradical capacities of Arazá byproducts (seeds and peel) have been evaluated using extracts prepared with various organic solvents, demonstrating the influence of solvent selection on antioxidant activity. Studies have shown an increase in the antioxidant capacity of methanolic extracts, which exhibited higher activity in ORAC and FRAP assays compared to aqueous extracts, correlating with phenolic content [8]. Conversely, research on ASs collected in the Colombian Andean region, extracted with different solvents, has indicated that the DPPH radical scavenging capacity is highest in the dichloromethane fraction, followed by the residual hydroalcoholic fraction and the ethanol extract [11]. These discrepancies underscore the critical role of solvent selection in extracting bioactive compounds from Arazá byproducts and the potential contribution of other compounds, such as carotenoids, particularly in medium-polarity solvents, such as dichloromethane. The variability in antioxidant capacity observed across studies highlights the complex interaction among solvent polarity, extraction efficiency, and the specific antioxidant assay employed.

Recently, given their biological properties, polyphenol-rich functional foods have been proposed as potential supplementary and nutraceutical treatments for diabetes mellitus. The inhibition of *α*-amylase and *α*-glucosidase, enzymes involved in carbohydrate digestion, regulates blood sugar levels and is a strategy for managing postprandial hyperglycemia (high blood sugar after meals), which is a common characteristic of diabetes [12]. Both enzymes are well-known therapeutic targets for the management of postprandial blood glucose levels. Various enzymatic inhibitors, such as acarbose, miglitol, and voglibose, have been found to be effective in targeting these enzymes. However, their use is associated with strong gastrointestinal side effects [13]. Consequently, the search for novel and safer inhibitors has become a significant area of research, prompting substantial interest in developing potent inhibitors from natural sources. Using natural products (especially polyphenols) to inhibit these enzymes represents a promising oral strategy for regulating carbohydrate metabolism and managing hyperglycemia [14].

Particularly, phenolic acids and their derivatives, such as p-hydroxybenzoic acid, show promise in inhibiting both *α*-amylase and *α*-glucosidase enzymes, which are crucial for carbohydrate digestion and glucose absorption. The effects of different substituents on the benzene ring of phenolic acids on *α*-amylase inhibition have been investigated through in vitro and molecular docking assays. Derivatives such as 2,3,4-trihydroxybenzoic acid exhibited the strongest inhibitory effect, indicating that a hydroxyl group at the 2-position on the benzene ring significantly enhances inhibitory activity, whereas methoxylation at the 2-position and hydroxylation at the 5-position have negative effects [15]. Additionally, the substrate type influences the inhibition, with solid starch substrates yielding more accurate results [16]. Compared to other polyphenols, benzoic acid derivatives demonstrate competitive inhibition, suggesting direct interaction with the enzyme’s active site [16]. EGCG, a catechin, also exhibits significant inhibitory effects on both *α*-glucosidase and *α*-amylase. EGCG inhibits *α*-glucosidase through non-competitive mechanisms [17]. The galloyl moiety in EGCG enhances its inhibitory capacity, making it more effective than epigallocatechin (EGC) [15]. EGCG, particularly in combination with chlorogenic acid, shows a synergistic effect on *α*-amylase inhibition [18]. Both benzoic acid derivatives and EGCG show potential in managing diabetes through enzyme inhibition, which slows carbohydrate digestion and glucose absorption. Therefore, given the high levels of EGCG and p-hydroxybenzoic acid in ASs, AS extracts could be utilized in the development of phytotherapeutics for diabetes management. Additionally, the ingestion of whole ASs could be considered to assess the synergistic effects of fiber consumption on carbohydrate absorption, complementing the effects of phenolic compounds. Future in vitro and in vivo studies are needed to demonstrate these effects and optimize the extracts for therapeutic use.

In addition, these findings suggest that the AS extract evaluated in this study contains phenolic compounds with potential anti-hyperlipidemic and anti-obesity effects that are achieved through mechanisms different from those directly assessed. Other studies have demonstrated that epicatechin gallate (ECG) and EGCG, which are common in green tea, can simultaneously inhibit mevalonate pathway enzymes such as mevalonate kinase (MVK), mevalonate 5-diphosphate decarboxylase (MDD), and farnesylpyrophosphate synthase (FPPS) [19]. Furthermore, molecular docking studies on these phenols have revealed favorable and stable interactions via hydrogen bonds with cellular receptors, as well as the inhibition of trimethylamine N-oxide (TMAO) production, providing novel insights into the mechanisms by which tea polyphenols prevent obesity [20].

However, more comprehensive evaluations are necessary to fully elucidate the effects of the phenolic compounds present and the impact of extraction methods on the recovery and concentration of specific phenolics. Finally, although a basic cytotoxicity assay was conducted, no cytotoxic effects were observed on blood cells, reinforcing the need for refined studies to understand the extract’s therapeutic potential on diabetes-related enzyme targets.

The antihypercholesterolemic activity was shown to be low (17.29 ± 0.01% inhibition). However, the extract also did not exhibit significant cytotoxic effects in the blood cell model, as the cell viability was 84.29% ± 0.4%, compared to 100% cell viability in the untreated control. In siliceous studies, it has been shown that EGCG, kaempferol, and other phenols can occupy the HMG-CoA binding site with low affinity and act as competitive inhibitors of substrate binding, thereby blocking nicotinamide adenine dinucleotide phosphate (NADP+) binding [21].

Collectively, the studies herein demonstrate the chemical and biological potential of Amazonian Arazá seeds, which are frequently discarded as byproducts. Consequently, further investigations involving extraction optimization, structure–activity relationships, in vivo and in silico assays, and comprehensive omics analyses are recommended to elucidate the properties of these seeds.

## 4. Materials and Methods

### 4.1. Reagents and Materials

The high-performance liquid chromatography (HPLC)-grade chemicals that were utilized in this research, including, methanol, water, and formic acid, were purchased from Merck. Also, various standard-grade compounds, including caffeine, theobromine, theophylline, (±)-catechin, (−)-epigallocatechin gallate (EGCG), (−)-epicatechin (EC), (−)-epicatechin gallate (ECG), (−)-epigallocatechin, caffeic acid, p-coumaric acid, rosmarinic acid, quercetin, naringenin, luteolin, kaempferol, pinocembrin, apigenin, cyanidin 3-rutinoside, and pelargonidin 3-glucoside were obtained from Sigma, a division of Merck, located in Darmstadt, Germany. Additionally, Antrone, GR for analysis, β-carotene synthetic, ≥93% (UV), powder, membrane-permeable yellow dye MTT (3-(4,5-dimethylthiazol-2-yl)-2,5-diphenyltetrazolium, bromide, *α*-glucosidase from *Saccharomyces cerevisiae*, and *α*-amylase from *Bacillus subtilis* were acquired from Sigma, a division of Merck, located in Darmstadt, Germany.

### 4.2. Extract Preparation

The ASs were obtained from byproducts of fruit processing companies in Florencia, Caquetá, Colombia. These byproducts were dried in an oven (UN-450, MEMMERT, Lingen, Germany) at 40 °C for 48 h and then ground. A 20 g sample of the ground seeds was subjected to ultrasound-assisted extraction in a 1:20 ratio of material to solvent. The extraction was performed in a 70:30 (*v*/*v*) ethanol/water solution using an ultrasonic processor at a power level of 50%. The sonication cycle consisted of 59 s pulses followed by 10 s rests, and the total extraction time was 15 min. The resulting extract was concentrated using a rotary evaporator (R300, BUCHI) to remove most of the ethanol and subsequently lyophilized in a Lyovapor L-200 (BUCHI) for future use.

### 4.3. Proximate and Mineral Seed Composition

The nutritional value (protein, fat, ash content, and dietary fiber) was determined according to AOAC procedures [22]. The macro-Kjeldahl method (N × 6.25) determined the protein content. The crude fat was determined using a Soxhlet apparatus, and the sample was extracted with petroleum ether. The ash content was estimated via incineration at 600 ± 15 °C for 5 h. The dietary fiber was analyzed using a gravimetric method. Mineral constituents comprising potassium (K), magnesium (Mg), iron (Fe), calcium (Ca), copper (Cu), manganese (Mn), zinc (Zn), and phosphorus (P) were determined using atomic absorption spectrophotometry (SHIMADZU AA-6300, Tokyo, Japan).

### 4.4. Chemical Characterization

The following compound groups were quantified through spectrophotometric methods using a Thermo Scientific™ Multiskan™ GO Microplate Spectrophotometer at the wavelengths specified for each method. The total carbohydrates were quantified using the Antrone method [23]. The total carotenoids were extracted with a hexane–acetone solution. β-carotene was used as a standard to create a calibration curve. The absorbance was measured at 450 nm, and the proteins were measured using the Bradford method with a BSA standard at 590 nm and 450 nm. Total phenols were determined, according to [24], using the Folin–Ciocalteu reagent and gallic acid as a standard. Finally, the flavonoids were measured at 510 nm with quercetin as a standard, using the test described by [25].

### 4.5. Analysis of Phenolic Compounds

The samples were prepared by dissolving them in a 1:1 methanol/water mixture containing 0.2% formic acid. This solution was then vortexed for 5 min and sonicated for 20 min. The prepared samples were analyzed using a Dionex Ultimate 3000 Ultra-High-Performance Liquid Chromatograph (UHPLC) from Thermo Scientific. This system featured a binary gradient pump, an automatic sample injector, and a column thermostat unit. For detection, an electrospray ionization (ESI) interface connected to a high-resolution Orbitrap mass spectrometer was used. The mass spectrometer was operated in positive mode at a capillary voltage of 3.5 kV. Chromatographic separation was achieved using a Hypersil GOLD Aq column (Thermo Scientific, Sunnyvale, CA, USA; 100 × 2.1 mm, 1.9 μm particle size). The mobile phase consisted of A, 0.1% (*v*/*v*) formic acid and 5 mM ammonium formate in water, and B, 0.1% (*v*/*v*) formic acid and 5 mM ammonium formate in methanol. The gradient elution started with 100% A, changing linearly to 100% B over 8 min, was held for 4 min at 100% B, and returned to the initial conditions in 1 min. The total run time was 13 min, with an additional 7 min post-run equilibration. The compounds were identified using full-scan acquisition mode and the extraction of ion currents (EIC) corresponding to the protonated molecules [M+H]^+^ of the compounds of interest, followed by fragmentation analysis. For quantification, calibration curves were generated using certified reference standards listed in the Reagents and Materials subsection.

### 4.6. DPPH Radical Cation Decolorization Assay

The methodology proposed by [26] was followed with some modifications. A 0.5 mL aliquot of a 0.02% DPPH solution in ethanol (96%) was added to 0.5 mL of each extract concentration. The mixture was stored in the dark (30 min), and the absorbance was measured at 517 nm against a blank (DPPH dissolved in ethanol). The percentage inhibition of DPPH was calculated using Equation (1):

(1) $$\%I=\frac{\left(A_{blank}-A_{Sample}\right)}{A_{blank}}\times 100$$

where A_blank_ is the absorbance of DPPH in ethanol, and A_sample_ is the absorbance of DPPH solution mixed with different extract concentrations. The IC50 value was defined as the effective concentration at which the DPPH radical was scavenged by 50%.

### 4.7. Biological Activities of the AS Extract

#### 4.7.1. Determination of Cell Viability Using an MTT Assay

After obtaining informed consent, blood samples were collected. Leukocytes were then isolated using the protocol described in reference [27]. To assess cell viability, a microplate assay was performed using four different concentrations of the extracts, each ranging from 156.25 to 1250 µL/mL. Each well contained 25 µL of leukocytes, 25 µL of extract, and 50 µL of MTT. These components were mixed and incubated at 37 °C for 1 h. Following incubation, DMSO [28] was added, and the mixture was stirred for 10 min to dissolve the formazan crystals. Finally, the absorbance of the microplates was read at 570 nm. The cell viability was determined for each sample in triplicate.

#### 4.7.2. Anticholesterolemic Activity

The activity of the HMG-CoA reductase enzyme was evaluated using a commercial kit from Sigma-Aldrich (N6505, St. Louis, MO, USA), following the manufacturer’s instructions. AS extracts were prepared at 25, 50, 100, 150, and 200 mg/mL concentrations to evaluate their inhibitory capacity on HMG-CoA reductase. Then, 180 µL of assay buffer and 20 µL of the HMG-CoA substrate solution were added to each well of a 96-well microplate. Next, 10 µL of HMG-CoA reductase enzyme was added to each well. Then, 10 µL of each extract concentration was added to the designated wells. The controls included 10 µL of pravastatin (positive control) and 10 µL of buffer (negative control). Reactions were initiated by adding 10 µL of NADPH (cofactor). Samples were incubated at 37 °C for 30 min. The enzymatic activity was assessed by monitoring NADPH oxidation via a spectrophotometric decrease in absorbance at 340 nm. The HMG-Co A reductase inhibition (%) was calculated using Equation (1).

#### 4.7.3. Inhibitory Activity of the α-Glucosidase Enzyme

The α-glucosidase enzyme (0.075 units) was mixed with the extract at different concentrations (50–200 μg/mL). Subsequently, 3 mM of p-nitrophenyl glucopyranoside (pNPG) was added as a substrate, and the reaction mixture was homogenized to initiate the reaction. The reaction mixture was incubated at 37 °C for 30 min, and the reaction was stopped by adding 2 mL of Na_2_CO_3_ [29]. The α-glucosidase activity was determined by measuring the release of p-nitrophenol from pNPG at 400 nm. The inhibitory activity of α-glucosidase was calculated using Equation (1).

#### 4.7.4. Inhibitory Activity of the α-Amylase Enzyme

The *α*-amylase enzyme was mixed with different concentrations of the extracts (50–200 μg/mL). A 0.5% starch solution was added as a substrate to initiate the reaction. The reaction mixture was incubated at 37 °C for 5 min and then terminated by adding 2 mL of DNS reagent (3,5 dinitrosalicylic acid). The reaction mixture was heated for 15 min at 100 °C and then diluted with 10 mL of distilled water in an ice bath [30]. Finally, the *α*-amylase activity was determined by measuring the absorbance at 540 nm [29]. The inhibitory activity of *α*-amylase was calculated using Equation (1).

### 4.8. Statistical Analysis

Dose–-concentration analyses were conducted using STATGRAPHICS Centurion XV, Version 15.2.06 software, employing non-linear simple regression models to determine the relationship between dose and response. The model that best fit the experimental data was selected to calculate the CI_50_, with its respective confidence interval also reported. The figures were generated using the Origin Pro 2023, Version 10.0.0.244 (SR1) statistical software package.

## 5. Conclusions

This research explored the nutritional and antioxidant potential of ASs, as well as its capacity to inhibit key enzymes associated with the control of glycemia and cholesterol levels, such as *α*-amylase, *α*-glucosidase, and HMG-CoA reductase. This byproduct is generated during the conversion of the fruit into pulp and, despite constituting a significant percentage of the fruit’s dry biomass, currently lacks valorization alternatives. Nutritionally, the results showed that the seeds primarily contribute a significant amount of fiber, which could be of interest for food applications; however, they exhibit low levels of lipids and protein. For the first time, significant levels of carotenes in the seeds were reported, approaching those reported for the peel and pulp. Furthermore, new evidence was provided regarding the combined inhibitory effect of the hydroethanolic extract on *α*-amylase and *α*-glucosidase enzymes, with a predominantly higher inhibition of *α*-amylase. Nevertheless, this combined activity could be advantageous for glycemic control and warrants in vivo evaluations of this effect. No appreciable effect on HMG-CoA reductase was observed, nor were cytotoxic effects on the blood cell model used. While previous reports in the literature exist regarding the antioxidant capacity of different AS extracts, the evidence suggests that this effect, as well as the observed inhibition of the aforementioned enzymes, is related to the high content of phenolic compounds, such as phenolic acids, and certain flavonoids, such as epicatechin gallate (ECG). However, due to potential variations in chemical composition resulting from agro-environmental factors in the fruit, further studies are necessary. Currently, the literature on this topic is limited; therefore, additional studies should be conducted to provide more detailed information. Furthermore, to increase the valorization of the *E. stipitata* species, these results can be used for future applications in the development of functional foods or supplements and the impact of agro-environmental factors on the concentrations of bioactive compounds.

## Figures and Tables

**Table 1:** Proximal characterization and spectrophotometric quantification of some metabolites in ASs.

Parameter Analyzed	Arazá Seeds
% Ash	0.82 ± 0.08
% Crude protein	4.96 ± 0.05
Ethereal extract	0.16 ± 0.02
% Crude fiber	27.7 ± 0.3
% Nitrogen	0.78 ± 0.05
% Ca	0.16 ± 0.02
% Mg	2.27 ± 0.2
Na (mg/kg)	1.02 ± 0.1
% K	0.01 ± 0.001
Fe (mg/kg)	20.2 ± 0.2
Cu (mg/kg)	0.07 ± 0.005
Mn (mg/kg)	21.6 ± 2.2
Zn (mg/kg)	14.0 ± 1.2
B (mg/kg)	5.51 ± 0.4
% P	13.5 ± 1.25
% S	<0.03
Carbohydrates (mg glucose/100 g d.w.)	13.80 ± 0.1
Total phenols (mg gallic acid/100 g d.w.)	155.88 ± 6.12
Flavonoids (mg quercetin/100 g d.w.)	41.68 ± 8.1
β-Carotenoids (mg β-carotene/100 g d.w.)	43.66 ± 1.5

**Table 2:** Phenolic composition of ASs.

Compound (mg/kg)	Rt (min)	LOQ (mg/kg)	Arazá Seeds
Theobromine	3.1	0.1	<0.1
Theophylline	3.7	0.1	<0.1
Epigallocatechin (EGC)	3.7	0.1	<0.1
Catechin	3.7	0.1	<0.1 *
Epicatechin (EC)	4.2	0.1	<0.1 *
p-Hydroxybenzoic Acid	3.8	0.4	1.1
Caffeine	4.1	0.1	<0.1
Caffeic Acid	4.2	0.1	<0.1
Vanillic Acid	4.6	0.1	<0.1
Epigallocatechin Gallate (EGCG)	4.1	0.2	<0.2
p-Coumaric Acid	4.7	0.1	0.9
Epicatechin Gallate (ECG)	4.6	0.1	2.3
Ferulic Acid	5.2	0.1	<0.1
Quercetin	5.8	0.1	0.4
Rosmarinic Acid	5.1	2.5	<2.5
Luteolin	6.0	0.1	0.2
Trans-Cinnamic Acid	5.8	0.4	<0.4
Naringenin	5.8	0.1	0.3
Apigenin	6.3	0.1	<0.1
Pinocembrin	6.6	0.1	0.1
Ursolic Acid	9.0	0.1	<0.1
Pelargonidin 3-glucoside	4.3	0.1	<0.1
Rutin	5.2	0.1	<0.1

Rt: retention time, LOQ: limit of quantification, * detected below the quantification level but above the detection level of the method. Quantification of the analytes was based on calibration curves constructed using the certified reference standards listed in the Reagents and Materials section.

**Table 3:** Non-linear regression models for the inhibition of DPPH, *α*-amylase, and *α*-glucosidase enzymes by the hydroethanolic extract of ASs and acarbose.

Assay	Regression Model	Correlation Coefficient	R^2^	IC50 (µg/mL)	CI 95% (µg/mL)
DPPH inhibition	%I = √(8099.05 – 261,116/Concentration)	−0.990	97.92%	46.64	(40.98–53.70)
ASs extract *α*-Amylase inhibition	%I = 1/(0.0104662 + 0.448657/Concentration)	0.916	84.01%	47.05	(33.42–60.56)
Acarbose *α*-Amylase inhibition	%I = √(−6499.51 + 2037.25 × ln(Concentration)	0.991	98.39%	82.88	(184.1–18.99)
ASs extract *α*-Glucosidase inhibition	%I = −5.2705 + 14.1288 × ln(Concentration)	0.994	98.94%	49.99	(61.81–39.98)
Acarbose *α*-Glucosidase inhibition	%I = √(9260.83 − 84,301.2/Concentration)	−0.928	84.92%	12.47	(9.18–15.74)

*p*-value ≥ 0.05 for all regression models at a 95% confidence level.

## Data Availability

Data are contained within the article.

## Abbreviations

AS	Arazá seeds
EGCG	Epicatechin gallate
EGC	Epigallocatechin
LC-ESI-LTQ-XL-MS/MS	Liquid Chromatography Electrospray Ionization Linear Ion Trap Mass Spectrometry
DPPH	2,2’-Diphenyl-1-1-picrylhydrazyl
CI	Confidence interval
HMG-CoA	3-Hydroxy-3-methylglutaryl coenzyme A
MVK	Mevalonate kinase
MDD	Mevalonate 5-diphosphate decarboxylase
FPPS	Farnesylpyrophosphate synthase
TMAO	Trimethylamine N-oxide
DMSO	Dimethyl sulfoxide
pNPG	*p*-Nitrophenyl glucopyranoside
DNS	3,5 Dinitrosalicylic acid
